# Leader Humility and Machiavellianism: Investigating the Effects on Followers’ Self-Interested and Prosocial Behaviors

**DOI:** 10.3389/fpsyg.2022.742546

**Published:** 2022-03-09

**Authors:** Shu-Chen Chen, Wen-Qian Zou, Na-Ting Liu

**Affiliations:** ^1^Ming Chuan University, Taipei, Taiwan; ^2^Ningbo Childhood Education College, Ningbo, China

**Keywords:** leader humility, Machiavellianism, sense of power, self-interested behavior, prosocial behavior

## Abstract

Existing research on leader humility primarily demonstrates its positive effects. This study challenges this view by proposing the potential negative effects of leader humility on followers’ behaviors. Furthermore, this paper employs the person-situation interactionist perspective to extend the research on integrating followers’ personality traits and leader humility. Specifically, this study proposed that leader humility triggers their followers’ sense of power; moreover, this study wagers that whether followers’ sense of power encourages self-interested or prosocial behavior in followers depends on their particular Machiavellian traits. The theoretical model was tested using the time-lagged supervisor–subordinate matched data obtained. Our findings revealed that follower Machiavellianism fosters the relationship between a sense of power and self-interested behavior but it weakens the relationship between a sense of power and prosocial behavior. Thus, this study provides a better understanding regarding the effect of follower personality and leader humility on follower behavioral reactions.

## Introduction

Humble leaders are known to positively affect their followers’ prosocial behavior; notably, a humble leader’s ethical behavior can influence their followers (Owens and Hekman, 2012). However, despite the ethical approach, leader humility may affect follower behavior in negative ways as well (Owens and Hekman, 2012). Studies have demonstrated that followers under humble leaders will act in their own interests and be indifferent to the common good (Qiuyun et al., 2020). Thus, to comprehensively understand the impact of leader humility on followers’ reactions and subsequent outcomes, both the positive and negative components of leader humility should be properly examined. Prosocial behaviors were defined as behaviors intended to benefit another individual, group, or organization (Ferris et al., 2011). Conversely, self-interested behaviors refer to actions that benefit oneself (Meglino and Korsgaard, 2004; DeCelles et al., 2012). However, the underlying mechanism of leader humility that triggers self-interested or prosocial behaviors remain unexplored. In addition, the boundary conditions facilitated by these underlying mechanisms need to be studied as well. In this study, we aimed to examine the effects of leader humility on followers’ self-interested or prosocial behaviors through followers’ sense of power, and the role of followers’ Machiavellianism as a moderator of this effect.

Few researchers argue that leader humility entails a good use of power, since it enables followers to rid themselves of “psychological hurdles,” which arise with a sense of power (Lin et al., 2019). Individuals possessing power become more likely to take advantage of others for their personal gain; furthermore, they display inappropriate behaviors as a result of viewing others as instruments (Magee and Galinsky, 2008). However, some studies have reported that power may result in positive treatment of others (Galinsky et al., 2003; Schmid Mast et al., 2009). Notably, existing literature on power does not decisively address the positive or negative effects of power (Guinote, 2007). Thus, it is important to examine the boundary conditions, which affect individual behaviors by impacting the individual’s perception of their own power. In this study, we employed a person-situation interactionist approach, which conceptualizes self-interested/prosocial behaviors as a joint function of personal and situational variables (Tett and Burnett, 2003). Additionally, this study adopted Machiavellianism as a moderator variable—due to its emphasized role in ethical behaviors in existing research—to examine the relationship between a sense of power and self-interested and prosocial behaviors. Individuals with high Machiavellianism primarily act according to self-interest, and they tend to employ deception and manipulation to achieve their goals (Sendjaya et al., 2016). Thus, individuals with a highly Machiavellian personality (i.e., high Machs) often specifically adhere to self-interest as their guiding principle (Dalton and Radtke, 2013; Sendjaya et al., 2016). High Machs view power as a tool to pursue their own interests rather than the common good and tend to engage in more self-interested behaviors than prosocial behaviors. Thus, we expect that followers who are high Mach will be more likely to take advantage of a leader’s humility because they will use the power given to them by a humble leader to serve their own interests.

**Figure 1:**
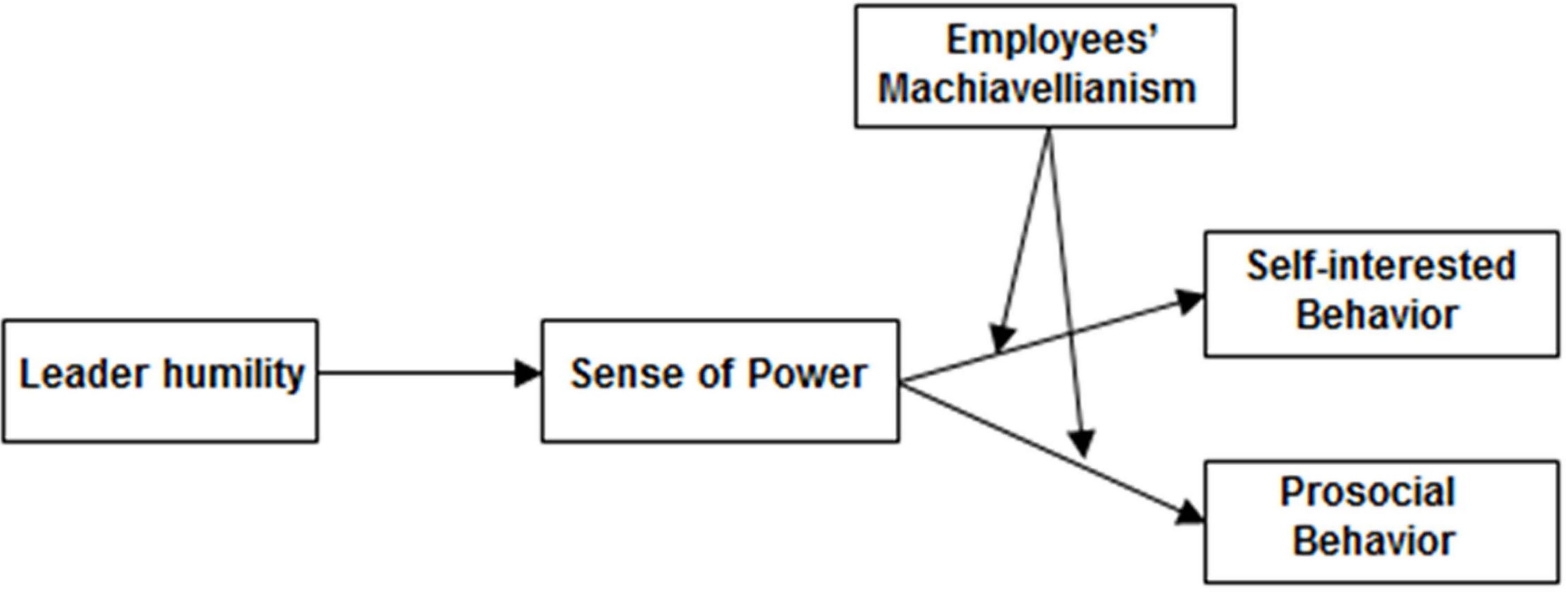
Theoretical model.

This study offers the following noteworthy insights. First, the existing empirical research indicates the positive or negative effect of leader humility on follower behavior. This study extended the existing literature by suggesting that leader humility can influence followers’ self-interested and prosocial behaviors. Moreover, this study addresses the need for further research on the underlying mechanisms and boundary conditions that facilitate the impact of leader humility on followers’ behaviors (Wang et al., 2018). Humble leaders display their vulnerability, appreciate their followers’ strengths and are willing to learn from them, and thereby de-emphasize hierarchy. Such behaviors signal followers regarding their potential significant influence on others (Anderson et al., 2012). Therefore, this study clarifies the relationship between leader humility and followers’ sense of power. Furthermore, the interactive effect of follower Machiavellianism and sense of power on their own self-interested and prosocial behaviors was examined through a person-situation interactionist approach. Figure 1 summarizes our model.

## Theory and Hypotheses

### Leader Humility and Sense of Power

There are various ways to define humility. Some definitions emphasize the intrapersonal, internal, and personal features of humility (Nielsen et al., 2010; Wright et al., 2018), while others have a clear external, expressed, or interpersonal focus (Morris et al., 2005; Owens et al., 2013; Ou et al., 2014). This study focuses on leader’s humble behaviors that emerges in interpersonal interactions and can be perceived by others. Hence, in this paper, we adopt Owens et al.’s (2013) framework of expressed humility. Expressed humility is defined as an interpersonal characteristic that emerges in social contexts and connotes (a) a willingness to view oneself accurately, (b) an appreciation of others’ strengths and contributions, and (c) teachability (Owens et al., 2013). In the current study, leader humility was measured through “expressed humility.” We argue that these three characteristics of leader humility are the key factors contributing to a follower’s enhanced personal sense of power. First, a humble leader projects transparency, which catalyzes a role-reversal process between themselves and their followers (Chiu et al., 2016). Leader humility follows a bottom-up model, which often involves practicing power-equalizing behaviors, where leaders delegate power toward others and away from themselves (Chiu et al., 2016). Such power-equalizing behaviors facilitate the removal of bureaucratic constraints to enable followers to enjoy freedom and a sense of power at work (Jeung and Yoon, 2018). Moreover, the humble leader’s appreciation of their followers’ strengths may, in turn, enhance their self-confidence. Thus, a humble leader’s followers are more likely to view their roles as more influential (Lin et al., 2019). Lastly, humble leaders tend to display more liberal attitudes toward others’ new or contradictory ideas, and they are more likely to incorporate their followers’ suggestions and comments (Owens et al., 2013; Argandona, 2015; Qian et al., 2018). Consequently, followers are more likely to experience a sense of power, due to their role in forming their humble leader’s decisions and perceptions. Thus, we developed the following hypothesis:

*Hypothesis 1*: Leader humility is positively related to followers’ sense of power.

### Followers’ Machiavellianism as a Boundary Condition

The approach–inhibition theory of power proposes that a sense of power can activate an approach system; it suggests that once individuals perceive themselves to be powerful, they become highly reward-driven and are keener to pursue personal interests, regardless of potentially conflicting interests (Keltner et al., 2003). This mindset allows them to focus on their own gains freely (Gruenfeld et al., 2008). In general, power widens the psychological and emotional distance from other people (Magee and Smith, 2013). Moreover, sense of power reduces one’s concern regarding others’ wellbeing or their social relationships, and it has been proven to promote individuals’ self-oriented behavior (Gruenfeld et al., 2008; Magee and Smith, 2013). Conversely, a growing body of literature indicates that power might lead people to put others’ needs before their own interests. For example, power may result in increased perspective-taking (Hall et al., 2005) and interpersonal sensitivity (Hall et al., 2009; Schmid Mast et al., 2009). Thus, followers’ sense of power had a significant double-edged sword effect on their reactions and subsequent outcomes. Furthermore, there is a need to better understand the boundary conditions, which facilitate or retard the performance of self-interested or prosocial behavior among followers with a sense of power.

Based on the effect of sense of power on followers’ prosocial or self-interested behaviors, some researchers have suggested that an individual’s underlying traits should be emphasized rather than the power directly affecting their behavior (Galinsky et al., 2003). Machiavellianism is popularly known for its association with power; it represents cynical views toward human nature and a cold, calculating attitude towards others. A higher degree of Machiavellianism can influence the decision-making process during ethical judgments (Schepers, 2003). Machiavellians aim to protect their self-interests, while striving to gain control over others and secure a privileged status for themselves; thus, this view is positively related to self-serving motives (Becker and O’Hair, 2007; Birkás et al., 2015). The decision-making process among highly Machiavellian individuals is largely guided by self-interest; thus, it can be argued that possessing a sense of power makes self-interested behavior more likely among highly Machiavellian individuals. Self-interested/prosocial behaviors were conceptualized as a function of personal and situational variables acting jointly to shape behavior. We argued that employees with high Machiavellianism display a tendency toward possessing a self-interested perspective. Such a perspective propels them to see power—to a higher extent—as a means to pursue their self-interests and achieve their personal objectives. Consequently, they opt for higher self-interested behavior and lower prosocial behavior. Thus, we hypothesized that:

*Hypothesis 2*: Follower Machiavellianism moderates the relationship between sense of power and self-interested behavior, such that this relationship is stronger when follower Machiavellianism is high rather than low.

*Hypothesis 3*: Follower Machiavellianism moderates the relationship between sense of power and prosocial behavior, such that this relationship is stronger when follower Machiavellianism is low rather than high.

### An Integrated Model

Humble leaders are open to: facing their weakness, recognizing their followers’ strengths, and incorporating their suggestions, which can foster followers’ senses of power. Moreover, we applied the person-situation interactionist approach and argued that a sense of power may not directly influence follower behavior, rather it interacts with follower Machiavellianism. Individuals high on Machiavellianism tend to interpret power from a self-interested perspective, resulting in the pursuit of their own goals over others’. Thus, we proposed that followers of humble leaders, with a higher personal sense of power, are expected to engage in more self-interested behaviors when Machiavellianism is high. However, high Machiavellians tend to be manipulative and calculating, with little or no concern for others’ welfare (Becker and O’Hair, 2007). We assume that followers of humble leaders, with a higher personal sense of power, are expected to engage in lower prosocial behaviors when they have high levels of Machiavellianism. Thus, we hypothesized that:

*Hypothesis 4*: Machiavellianism moderates the indirect effect of leader humility on a follower’s self-interested behaviors through their personal sense of power, such that the indirect effect is stronger when the follower’s Machiavellianism is high.

*Hypothesis 5*: Machiavellianism moderates the indirect effect of leader humility on a follower’s prosocial behaviors through their personal sense of power, such that the indirect effect is weaker when the follower’s Machiavellianism is high.

## Materials and Methods

### Participants and Procedure

In this study, we compiled data collected from 31 Chinese companies. First, we contacted the directors of each company and explained the objectives of this study before distributing our questionnaires. Moreover, multi-wave and multisource data were obtained to reduce common method variance. We designed two sets of questionnaires as per the role of the respondents: (1) subordinates (followers) and (2) supervisors (leaders). Followers were asked to assess their leaders’ levels of humility and their own Machiavellian traits at Time 1 of data collection. At Time 2 (1 month after Time 1), followers and supervisors were provided the questionnaires separately. Followers were asked to assess their personal sense of power and self-interested behaviors, while supervisors were asked to evaluate their followers’ prosocial behaviors. A researcher-assigned identification number was encoded with each questionnaire to match followers’ responses to their corresponding supervisors’. Each respondent was instructed to directly return the questionnaires after sealing them in the envelopes, and a gift (worth approximately USD 3.00) was offered to them.

This study has addressed and taken precautions to reduce social desirability bias (Podsakoff et al., 2012). First, participants were informed that their questionnaire responses were completely anonymous, the results of this research would be used for academic purposes only, and all data would be kept confidential (Nederhof, 1985; Randall and Fernandes, 1991; Flannery and May, 2000). Secondly, after completing the questionnaires individually, the participants put the completed questionnaires into sealed envelopes and handed them directly to the researcher (Grau et al., 2019). Above precautions were used to make ensure that respondents can fill out the answers with confidence while privacy was fully guaranteed.

A total of 330 questionnaires were distributed, and 241 valid pairs (return rate: 73.03%) were obtained after excluding incomplete questionnaires. The sample of supervisors and subordinates included a female population of 74.30% and 61.40%, with an average age of 42.34 years (*SD* = 5.43) and 29.51 years (*SD* = 7.06), and 86.30% and 90.90% with a bachelor’s degree, respectively.

### Measures

In this study, all scales employed were adapted into Chinese, using the “translation/back-translation” methodology. Each scale was rated on a 7-point Likert scale, ranging from 1 (*strongly disagree*) to 7 (*strongly agree*).

#### Leader Humility

Leader humility was assessed by adopting the 9-item scale of Owens et al. (2013) on expressed humility. A sample item included “My supervisor acknowledges when others have more knowledge and skills than him- or herself” (*α* = 0.93).

#### Personal Sense of Power

Personal sense of power was assessed by adopting the 8-item scale of Anderson et al. (2012). A sample item included “I can get him/her/them to listen to what I say” (*α* = 0.76).

#### Machiavellianism

Machiavellianism was measured using the 5-item scale of Valentine and Fleischman’s (2003) based on the 20-item Mach-IV scale (Christie and Geis, 1970). Existing literature has confirmed that this 5-item measure is highly correlated the original 20-item measure (*r* = 0.80, *p* < 0.001; Zagenczyk et al., 2011). A sample item included “The best way to handle people is to tell them what they want to hear” (*α* = 0.76).

#### Self-Interested Behavior

In this study, we measured certain forms of self-interested behavior as per the priority of their disadvantage to the company, as discussed by DeCelles et al. (2012). This concept corresponds to behaviors categorized as organizational deviance (Aquino et al., 1999; Bennett and Robinson, 2000). For this purpose, we selected three items on organizational deviance (Aquino et al., 1999) focusing on the intentional avoidance of work while still getting paid. This sheds light on followers’ self-interested behaviors. A sample item included “I lied about the number of hours that I worked” (*α* = 0.92).

### Prosocial Behavior

Previous studies have used helping behaviors as an indicator of focal prosocial behavior (Balliet and Ferris, 2013; Hafenbrack et al., 2020). For this purpose, we have adapted the 4-item scale of Coleman and Borman (2000) on interpersonal helping behaviors to evaluate the outcomes of prosocial behaviors. Accordingly, leaders rated the focal participants’ engagement in several interpersonal helping behaviors. A sample item was “This person is engaging in behavior that benefits individuals in the organization” (*α* = 0.90).

#### Control Variables

Considering that demographic variables influence employees’ work attitudes and performance (Van Der Vegt and Bunderson, 2005; Lam et al., 2015), we controlled for followers’ gender (0 = male, 1 = female), age (in years), education (1 = junior college or under, 2 = bachelor’s degree, 3 = master’s degree or higher). In addition, demographic characteristics of leaders may affect their cognitive styles and decision-making (Hambrick and Mason, 1984), thus affecting their subordinates’ perceptions, attitudes, and behaviors. Therefore, we controlled for leaders’ gender (0 = male, 1 = female), age (in years), education (1 = junior college or under, 2 = bachelor’s degree, 3 = master’s degree or higher). Finally, we also controlled for dyadic tenure (Chen et al., 2019), which was defined as “the period of time during which the supervisor and followers worked together.” The followers reported how long (in years) they have worked with their supervisors.

### Statistical Analysis

All collected data were analyzed using Mplus 7.2. Participants were divided into 35 teams, where each leader rated their corresponding followers’ prosocial behavior. Each participant’s data were nested within a supervisory unit with other participants’ data. Thus, we used the “Cluster” and “Type = Complex” Mplus syntax to account for the non-independence of the data. Moreover, we employed the Monte Carlo method to estimate the confidence intervals for indirect effects to test our mediation hypotheses.

## Results

### Confirmatory Factor Analysis

We performed a confirmatory factor analysis to examine the proposed five-factor model fit to the data. Moreover, four alternative models were constructed in addition to the hypothesized five-factor model (Table 1). The results indicated that the five-factor model is substantially superior to the other constructed models (*χ*^2^ = 705.01, df = 364, *χ*^2^/df = 1.94, CFI = 0.91, TLI = 0.90, RMSEA = 0.06). Table 2 presents the study’s descriptive statistics, findings, and analyses.

**Table 1:** Confirmatory factor analysis of variables.

Model	Factors	χ^2^	dƒ	χ^2^/dƒ	CFI	TLI	RMSEA	Δχ^2^	Δdƒ
1	5-factor: LH; SP; SB; PB; EM	705.01	364	1.94	0.91	0.90	0.06		
2	4-factor; LH + SP; SB; PB; EM	1392.03	371	3.75	0.74	0.72	0.11	687.02^***^	7
3	3-factor; LH + SP + EM; SB; PB	1610.40	374	4.31	0.68	0.66	0.12	905.39^***^	10
4	2-factor; LH + EM + PB; SP + SB	2399.01	376	6.38	0.48	0.44	0.15	1694.00^***^	12
5	1-factor; LH + SP + SB + PB + EM	2856.97	377	7.58	0.37	0.32	0.16	2151.96^***^	13

*LH: Leader humility; SP: Sense of power; SB: Self-interested behavior; PB: Prosocial behavior; EM: Employee Mach; CFI: comparative fit index; TLI: Tucker–Lewis index; RMSEA: root mean square error of approximation*.

*** *p < 0.001*.

**Table 2:** Means, standard deviations and correlations (*N* = 241).

	1	2	3	4	5	6	7	8	9	10	11	12
1. Leader age	__											
2. Leader gender	0.21^**^	__										
3. Leader education	0.12	−0.33^**^	__									
4. Follower age	0.063	−0.25^*^	−0.04	__								
5. Follower gender	−0.03	−0.06	0.03	0.00	__							
6. Follower education	−0.05	−0.05	0.17^**^	−0.12	−0.01	__						
7. Dyadic tenure	0.24^**^	0.23^**^	−0.06	0.38^**^	−0.04	−0.10	__					
8. Leader humility	0.06	0.23^**^	−0.10	−0.16^*^	−0.03	0.01	−0.06	(0.93)				
9. Sense of power	−0.08	0.08	−0.10	−0.09	0.04	0.15*	−0.06	0.15^*^	(0.76)			
10. Self-interested	−0.10	−0.14^*^	−0.10	0.10	0.13*	−0.01	0.00	−0.01	0.28^**^	(0.92)		
11. Prosocial behavior	−0.14^*^	0.34^**^	−0.20^**^	−0.08	0.10	0.04	0.12	0.09	0.21^**^	−0.063	(0.90)	
12. Employee Mach	0.010	−0.23^**^	0.054	0.15*	−0.09	−0.03	0.02	−0.31^**^	−0.17^**^	0.11	−0.23^**^	(0.76)
Mean	42.34	1.74	2.09	29.51	1.61	1.98	3.26	4.69	3.53	1.41	4.59	2.83
SD	5.43	0.44	0.36	7.06	0.49	0.30	3.02	0.88	0.49	0.86	0.85	0.88

*Gender was coded 1 = female, 0 = male. Education was coded 1 = junior college or under, 2 = bachelor’s degree, 3 = master’s degree or higher. Cronbach’s alphas are on the diagonal in parentheses*.

* *p < 0.05*;

** *p < 0.01*.

### Hypotheses Testing

As shown in Table 3, Model 1 revealed that leader humility is positively related to sense of power (*β* = 0.14, *p* < 0.05). Thus, Hypothesis 1 was supported.

**Table 3:** Results of hierarchal regression.

Variable	Sense of power	Self-interested behavior		Prosocial behavior	
	Model 1	Model 2	Model3	Model 4	Model 5
Leader age	−0.07 (0.27)	−0.04 (0.58)	−0.04 (0.57)	−0.21^*^ (0.04)	−0.21^*^ (0.04)
Leader gender	0.03 (0.75)	−0.16 (0.32)	−0.16 (0.33)	0.32^*^ (0.01)	0.32^*^ (0.01)
Leader education	−0.10 (0.12)	−0.12 (0.28)	−0.12 (0.29)	−0.05 (0.64)	−0.05 (0.64)
Follower age	−0.04 (0.69)	0.05 (0.42)	0.05 (0.40)	0.01 (0.91)	0.01 (0.92)
Follower gender	0.05 (0.30)	0.13^**^ (0.01)	0.13^**^ (0.01)	0.09 (0.10)	0.08 (0.09)
Follower education	0.15^**^ (0.01)	−0.04 (0.63)	−0.04 (0.63)	0.05 (0.48)	0.05 (0.48)
Dyadic tenure	−0.01 (0.84)	0.02 (0.72)	0.02 (0.71)	0.13 (0.21)	0.13 (0.20)
Leader humility	0.14^*^ (0.04)		0.01 (0.88)		−0.01 (0.95)
Sense of power(SP)		0.28^**^ (0.00)	0.28^**^ (0.00)	0.17^**^ (0.00)	0.17^**^ (0.01)
Employee Mach(EM)		0.14 (0.10)	0.14 (0.07)	−0.13 (0.07)	−0.13 (0.09)
SP× EM		0.15 ^*^ (0.03)	0.15^*^ (0.03)	−0.18^*^ (0.03)	−0.18^*^ (0.03)
R^2^	0.07* * (0.00)	0.18 ^*^ (0.01)	0.18^*^ (0.01)	0.26 ^**^ (0.00)	0.26 ^**^ (0.00)

*Values in parentheses represent p-values*.

* *p < 0.05*;

** *p < 0.01*.

**Figure 2:**
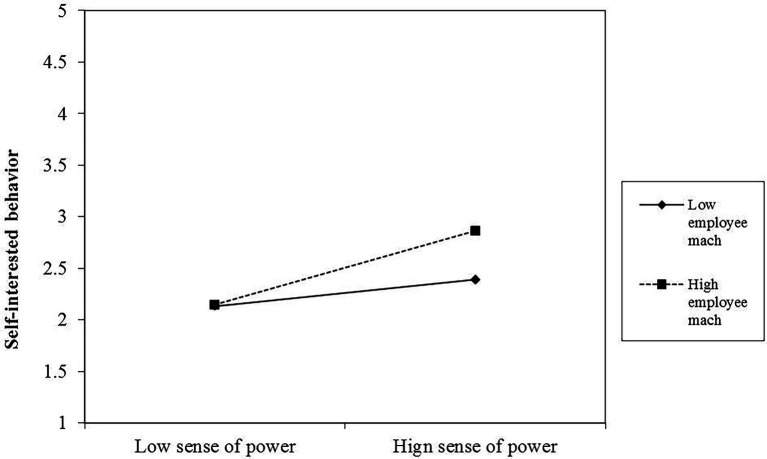
Moderating effect of followers’ Machiavellianism between a sense of power and self-interested behavior.

**Figure 3:**
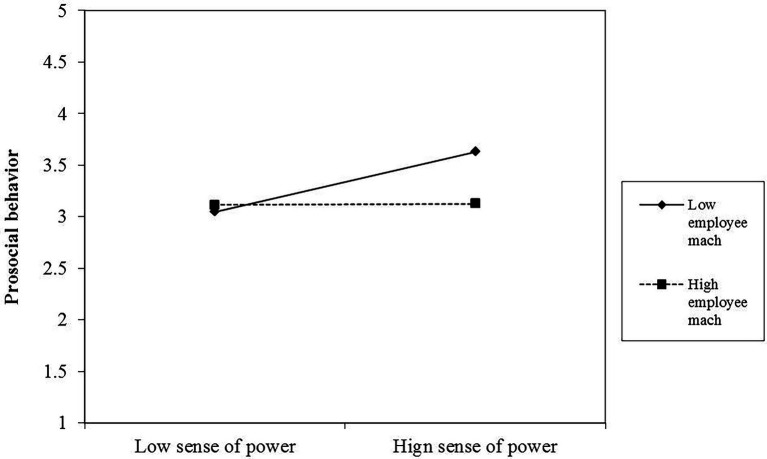
Moderating effect of followers’ Machiavellianism between a sense of power and prosocial behavior.

Table 3 presents the interaction between sense of power and followers’ Machiavellianism, suggesting that this interaction has a significant effect on self-interested (Model 2: *β* = 0.15, *p* < 0.05) and prosocial behavior (Model 4: *β* = −0.18, *p* < 0.05). Moreover, the relationships between sense of power, self-interested behavior, and prosocial behavior were plotted at both high and low follower Machiavellianism (1 SD above and below the mean). As illustrated in Figure 2, the simple slope test reported a more positive relationship between sense of power and followers’ self-interested behavior, when followers’ Machiavellianism was high (*β* = 0.74, *p* < 0.01) rather than when it was low (*β* = 0.26, *ns*). Therefore, Hypothesis 2 was supported. Accordingly, the simple slope test in Figure 3 reported a more positive relationship between sense of power and followers’ prosocial behavior when followers’ Machiavellianism was low (*β* = 0.59, *p* < 0.01) rather than when it was high (*β* = 0.01, *ns*). Therefore, Hypothesis 3 was supported.

Lastly, we tested the moderated mediation model proposed in Hypotheses 4 and 5 using 20,000 Monte Carlo replications. The results reported that leader humility indirectly affects self-interested behavior *via* sense of power; this effect is stronger when follower Machiavellianism is higher (indirect effect = 0.06, 95% Confidence Interval [CI] [0.005, 0.130]), than when it is lower (indirect effect = 0.02, 95% CI [−0.006, 0.064]). Moreover, there is a significant difference between the indirect effect generated by high and low Machiavellianism (indirect effect difference = 0.04, 95% CI [0.001, 0.092]). However, the indirect effect of leader humility on prosocial behavior is stronger *via* sense of power when follower Machiavellianism is lower (indirect effect = 0.04, 95% CI [0.003, 0.118]), than when it is higher (indirect effect = 0.00, 95% CI [−0.020, 0.016]). Additionally, there is also a significant difference between the indirect effect of high and low Machiavellianism (indirect effect difference = −0.04, 95% CI [−0.128, −0.001]). Thus, Hypotheses 4 and 5 are supported.

## Discussion

Our study focuses on the effect of leader humility on followers’ self-interested and prosocial behaviors. In accordance with our hypotheses, this study demonstrated that leader humility triggers followers’ sense of power. Furthermore, this study clarified that whether this sense of power encourages self-interested or prosocial behavior in followers is dependent on the Machiavellian traits of those followers. Specifically, followers who are high Mach will be more likely to take advantage of a leader’s humility because they will use the power given to them by a humble leader to serve their own interests.

### Theoretical Implications

Humility is an important virtue, which can influence positive outcomes among followers. However, our study challenges this notion regarding the positive effects of leader humility (Argandona, 2015; Owens et al., 2015; Rego and Simpson, 2018). In line with the findings of Qiuyun et al. (2020), leaders are recommended to hold a more nuanced view of humility and address the advantageous as well as the disadvantageous effects of leader humility. Humble leaders must manage non-uniformity, while seeking an optimal point along the continuum, to maximize the positive effects of their leadership style (Tsoukas, 2018). Our study extended the literature by demonstrating both the positive (prosocial behavior) and negative (self-interested) components arising from leader humility.

Moreover, numerous research studies have confirmed that followers may differ in their responses to leadership, and this difference is largely dependent on their personality traits (Belschak et al., 2015, 2018). In this study, we applied the person-situation interactionist approach and demonstrated the key role of Machiavellianism in forming an individual’s self-interested/prosocial behaviors. Anderson et al. (2012) emphasized the need for further research on personal sense of power by examining the boundary conditions. For this purpose, we focused on followers’ Machiavellianism, to examine how this trait moderates the effect of sense of power on followers’ self-interested/prosocial behaviors. Lastly, our findings indicated that Machiavellianism has an indirect moderating effect on followers’ self-interested/prosocial behaviors through their sense of power. Our research findings confirmed that a humble leader alleviates their followers’ senses of power (Qiuyun et al., 2020); however, higher sense of power serves as a double-edged sword with different effects on followers’ behaviors. Therefore, we developed an integrated model to examine the interaction between a follower’s sense of power and their level of Machiavellianism, which enabled us to observe its functions as an underlying mechanism of the contradictory behaviors—self-interested and prosocial—demonstrated among followers. Thus, this study deepens our understanding regarding the effects of leader humility on followers’ behaviors through the person-situation interactionist approach.

### Practical Implications

Leader humility is favorable due to its potential to generate beneficial effects among subordinates as well as organizations. However, our current findings suggest that leader humility may trigger followers’ unfavorable behaviors. Furthermore, a sense of power promotes a follower with a low level of Machiavellianism to engage in more prosocial behaviors; however, a sense of power leads a follower with a high level of Machiavellianism to engage in more self-interested behaviors. Thus, practitioners should focus on followers’ Machiavellianism traits. This study provides implications for organizations by suggesting the need to measure applicants’ levels of Machiavellianism during recruitment procedures. In addition, organizations can reduce the negative consequences of Machiavellianism by issuing explicit policies to outline clear guidelines for acceptable behaviors within their premises. Lastly, leader humility is expressed through a leader’s understanding of his or her subordinates’ personalities. Leaders can display higher humility among followers with lower levels of Machiavellianism and vice versa.

### Research Limitations and Suggestions

This study does have certain limitations. Despite taking precautions to reduce social desirability bias, it is not possible to completely eliminate its effects. Future studies should combine self-report measures with third-party reporting for variables such as self-interested behavior, or make social desirability a control variable to mitigate the social desirability bias caused by self-report methods (Umphress et al., 2010). Moreover, despite the multiple sources and varied time periods of our data collection, our research design restricted our findings in terms of establishing causality. Thus, in order to confirm the causal relationship between the study variables, future studies should employ a longitudinal research design. Thirdly, all research samples in this study are located within Chinese contexts; therefore, further research is needed in other cultural settings to verify the generalizability of our findings.

## Data Availability Statement

The datasets generated and analyzed during the current study are not publicly available due to privacy reasons, but are available from the corresponding author on reasonable request. Requests to access these datasets should be directed to S-CC, chchen1005@hotmail.com.

## Ethics Statement

All procedures performed in studies involving human participants were in accordance with the ethical standards of the institutional and/or national research committee and with the 1964 Declaration of Helsinki and its later amendments or comparable ethical standards. The patients/participants provided their written informed consent to participate in this study.

## Author Contributions

S-CC acted as the principal investigator and oversaw the study from its inception to completion. W-QZ was responsible for data collection, writing the manuscript, and conceptualizing the models. N-TL contributed to rewriting the subsequent drafts of the paper after the initial submission. All authors contributed to the article and approved the submitted version.

## Funding

This study was financially supported by Ministry of Science and Technology, Taiwan.

## Conflict of Interest

The authors declare that the research was conducted in the absence of any commercial or financial relationships that could be construed as a potential conflict of interest.

## Publisher’s Note

All claims expressed in this article are solely those of the authors and do not necessarily represent those of their affiliated organizations, or those of the publisher, the editors and the reviewers. Any product that may be evaluated in this article, or claim that may be made by its manufacturer, is not guaranteed or endorsed by the publisher.
